# Beyond Knowledge and Attitude: Structural Drivers Dominate Canine Rabies Vaccination Decisions in Rural China: A Multilevel Modeling Study

**DOI:** 10.1155/tbed/4901283

**Published:** 2026-10-21

**Authors:** Kun Sun, Shuilian Chen, Chenlin Duan, Jinsong Qiu, Hong Feng, Peng Zhang, Gangdeng Zheng, Yinzhu Zhou, Qiangming Xie

**Affiliations:** ^1^ Changsha Center for Disease Control and Prevention (Changsha Health Comprehensive Supervision and Law Enforcement Bureau) 509 Wanjiali North Road Changsha Hunan Province China; ^2^ Liuyang Center for Disease Control and Prevention 11 Lihua Road Changsha Hunan Province China; ^3^ Ningxiang Center for Disease Control and Prevention 249 Chuncheng North Road Changsha Hunan Province China; ^4^ Changsha County Center for Disease Control and Prevention 96 Teli Road Changsha Hunan Province China

**Keywords:** KAP, rabies, rural areas, structural factors, vaccination of dogs

## Abstract

The knowledge–attitude–practice (KAP) model guides rabies health promotion but insufficiently explains the knowledge‐practice gap. Existing research largely focuses on individual cognitive factors, lacking integration of multilevel and structural determinants. This study is based on multistage sampling and conducts a cross‐sectional survey of rural populations. The multilevel mixed effects model and structural equation model were used to analyze the KAP path of the general population, and a binomial mixed effects model was used to analyze the factors affecting dog vaccination in dog‐owning households. A total of 2148 rural residents were included in the study. The KAP analysis revealed a moderate mean knowledge score (68.04 ± 22.24) and a high proportion of positive attitudes (88.13%), the active practice rate was low (26.53%). Attitude emerged as a significant mediator, through which knowledge positively influenced (*β* = 0.12, *p* < 0.001). The indirect effect accounts for 91.04% of the overall. An analysis of 282 dog‐owning households found that the vaccination rate for dogs was 36.17%. The mixed effects model showed that the vaccination rate of purchased dogs was 3.38 times higher than that of home‐bred dogs (OR = 3.38, 95% CI: 1.53–7.45), and the vaccination rate of leash‐restrained dogs was 3.77 times higher than that of free‐ranging dogs (OR = 3.77, 95% CI: 1.84–7.72). In addition to knowledge and attitude, structural factors such as dog sources and dog management methods significantly affect dog vaccination. Rabies prevention should evolve from education toward comprehensive interventions that include dog management reforms, and the creation of supportive policy and environments that reduce structural barriers.

**Summary**

• Health education alone is insufficient to change rabies prevention behaviors; addressing structural barriers such as uncontrolled dog roaming is essential.
• Integrating multilevel data reveals that dog sources and dog management methods are stronger predictors of vaccination than knowledge and attitude.
• Our findings urge rabies prevention evolve from education toward comprehensive interventions that include dog management reforms and the creation of supportive policy and environments that reduce structural barriers.

## 1. Introduction

With a case fatality rate nearly 100%, rabies constitutes an enduring threat to the population that is disproportionately concentrated in Asia and Africa [1, 2]. Targeting the canine reservoir for control and elimination efforts represents a fundamental strategy for rabies prevention, given that dog‑mediated transmission accounts for over 95% of human cases [3]. Without a canine vaccination coverage exceeding 70%, the goal of eliminating human rabies is unlikely to be attained [3, 4]. Hence, intervention programmes must prioritize dog owners’ compliance with two key actions: vaccinating their animals and seeking post‐exposure prophylaxis (PEP) without delay after a suspected exposure.

As a heuristic framework for health behaviors, the knowledge–attitude–practice (KAP) model holds that knowledge shapes attitudes, and these attitudes subsequently encourage protective practices [5, 6]. Rabies studies have often adopted it for surveys and education [7–9]. However, the repeated “knowledge‐practice gap” suggests that improved better knowledge does not necessarily translate into vaccination or timely care [10–12], warranting a more nuanced evaluation of its actual utility.

Current KAP‐based rabies research has several limitations: most overemphasize individual cognition, with insufficient attention to structural factors like dog management policies, veterinary access, and vaccination drives that affect coverage independently of awareness [13–15]; conventional regression models used in cross‐sectional designs ignore households/communities nesting, leading to biased errors and dubious inferences, whereas mixed‐effect models offer a better fit [16, 17]; and the literature tends to be fragmented, either testing the KAP pathway in general populations or exploring single dog‑owner traits, without a unified framework that jointly assesses theoretical hypotheses and uncovers subgroup specific obstacles.

To bridge these gaps, we undertook a broad survey across rural Changsha, China, pursuing two objectives: (i) rigorously test the classical KAP pathway in the general population and (ii) identify broader, potentially dominant real world drivers of dog vaccination specifically among dog owners, using multilevel and multigroup analytical strategies. This design should sharpen our insights into where rabies control efforts could be most effectively targeted.

## 2. Materials and Methods

### 2.1. Research Design and Sample

Between August and October 2024, we conducted a cross‐sectional survey in the rural areas of Changsha, Hunan, China. Participants were recruited via a multistage random sampling scheme: from each of the three administrative regions (Changsha County, Liuyang City, and Ningxiang City), at least five townships were randomly drawn; from each township, at least 50 households were randomly selected; and within each household, all eligible and accessible members were invited. Our target was ≥650 respondents per county. Eligible individuals were those aged ≥14 years, who had lived locally for ≥3 months, and who could give informed consent and complete the survey with adequate cognitive capacity.

We set the per county minimum sample size using the rabies core knowledge awareness rate as the primary outcome. Drawing on recent surveys in rural China, we assumed that the rate be approximately 60% (*p* = 0.60) [18]. With the 95% confidence level *α* = 0.05(*Z* = 1.96) and an absolute allowable error *d* = 0.04, the standard cross‐sectional formula gave *n*_0_ = *Z* ^2^ *p*(1−*p*)/*d* ^2^ = 576. Allowing for 10% nonresponse, the adjusted sample size per county came to 576/0.9 ≈ 640. We therefore set the sample size per county at 650, a number that also meets the precision demands for secondary outcomes. In the end, 2148 participants were actually enrolled, which further bolsters the stability of our estimates.

### 2.2. Operational Definitions

We operationally defined leash‐restrained dogs as those temporarily restrained by a leash or chain attached to a stationary object while outdoors and directly watched by the owner, a restriction imposed specifically to prevent bites or scratches. This practice does not constitute prolonged, unsupervised tethering and is distinct from permanent confinement that raises animal welfare concerns. Free‐ranging dogs were defined as dogs that are allowed to roam outdoors without a leash and without direct owner supervision, regardless of whether they are owned or community dogs. This includes both dogs that roam freely for part of the day and those that are unrestrained full‐time.

### 2.3. Data Collection

Trained disease control investigators administered a face‐to‐face questionnaire survey covering rabies knowledge, attitudes, and practices. Data were entered and verified via double‐entry verification. The full list of items is given in Table S1.

### 2.4. Data Analysis

To correct for potential bias between the sample and the overall demographic distribution, we applied a post‐stratification weighting method. The weights were calibrated based on the joint distribution of samples across counties and the age‑by‑gender cross‑classification. These weights were applied in all subsequent descriptive statistics and model inferences.

All domain scores (knowledge, attitude, and practice) were standardized to a continuous 0–100 scale. Knowledge was treated as a continuous variable in subsequent analyses, whereas attitude and practice were dichotomized into positive (score ≥85) and non‑positive (<85) categories for binary regression modeling.

For the general population, the KAP analysis accounted for the multilevel data structure, in which individuals were nested within households, households within townships, and townships within counties. Given that only three counties (including county‐level cities) were available, which was insufficient to stably estimate the random effect variance at the county level, a three‐level mixed effects model was ultimately fitted, with individuals at Level 1, households at Level 2, and townships at Level 3. Specifically, the knowledge score, a continuous variable, was analyzed using a linear mixed model; attitude and practice, both binary variables, were analyzed using generalized linear mixed models with a binomial distribution. The mediating pathways among KAP were examined via structural equation modeling.

To identify factors associated with dog vaccination, we extracted the subset of dog owning households from the general population. To avoid pseudo‐replication and estimation bias due to household‐level clustering, the household was designated as the unit of analysis. From each dog owning household, we randomly drew one respondent, which gave us 282 households for current analysis. Having one observation per household kept the data points independent and supported valid statistical inference. For dog vaccination status, we fitted a binomial generalized linear mixed model with township‐level random intercepts to adjust for regional clustering.

All analyses were performed in R 4.5.2, relying on the survey, lme4, lmerTest, lavaan, and performance packages. Means and standard deviations were used for continuous variables, while frequencies and percentages for categorical variables. The threshold for statistical significance was predefined at *α* = 0.05 for all tests.

## 3. Results

### 3.1. Basic Information

The study enrolled 2148 rural residents, with males accounting for 47.77% and the 36–59 age group representing 45.44%. The KAP profile revealed a marked discrepancy: knowledge score was moderate (68.04 ± 22.24), attitudes were highly positive (88.13%), but active practice was strikingly low (26.53%). Univariate subgroup analyses (Table 1) further detailed this pattern. Gender did not significantly affect any dimensions (all *p* > 0.05). Age and education correlated with knowledge and attitude, yet they did not translate into significant practice differences across subgroups (*p* > 0.05). By contrast, household income strongly predicted practice, with higher‐income groups reporting more active practices (*p* < 0.05). Higher knowledge also linked to better attitudes and higher practice rates (all *p* < 0.05). However, even among those with higher knowledge or income, the active practice rates stayed far below the attitude levels, indicating a persistent KAP gap observable across almost all strata.

**Table 1:** Basic information and univariate analysis of rabies KAP among rural residents.

Variables	Classifications	Cases, *n* (%)	Knowledge			Attitude			Practice		
			Score (*x̄* ± *s*)	*F*/*t*	*p*‐Value	Positivity rate (%)	*χ* ^2^	*p*‐Value	Activity rate (%)	*χ* ^2^	*p*‐Value
County	Changsha	696 (32.40)	70.56 ± 21.06	7.44	0.006	92.64	9.17	<0.001	29.17	19.36	<0.001
	Liuyang	724 (33.71)	66.72 ± 22.22	—	—	86.85	—	—	33.72	—	—
	Ningxiang	728 (33.89)	66.77 ± 23.29	—	—	84.12	—	—	15.52	—	—
Gender	Male	1026 (47.77)	68.28 ± 21.61	−0.43	0.667	86.84	2.27	0.132	26.30	0.05	0.831
	Female	1122 (52.23)	67.80 ± 22.88	—	—	89.18	—	—	26.74	—	—
Age groups	14–17	187 (8.71)	62.07 ± 23.99	23.13	<0.001	83.77	17.32	<0.001	19.09	1.59	0.192
	18–35	485 (22.57)	72.01 ± 20.40	—	—	92.91	—	—	27.07	—	—
	36–59	976 (45.44)	71.23 ± 19.50	—	—	90.68	—	—	25.64	—	—
	≥60	500 (23.28)	59.02 ± 25.63	—	—	78.38	—	—	29.35	—	—
Education	Primary school and below	375 (17.46)	57.10 ± 27.22	117.80	<0.001	76.51	22.51	<0.001	29.67	1.02	0.382
	Middle school/high school/vocational school	1205 (56.10)	67.40 ± 21.25	—	—	87.45	—	—	25.07	—	—
	Associate degree	278 (12.94)	75.89 ± 15.58	—	—	97.01	—	—	28.95	—	—
	Bachelor’s degree or above	290 (13.50)	76.92 ± 17.88	—	—	95.79	—	—	25.99	—	—
Household size	1–2	122 (5.68)	64.11 ± 25.83	1.00	0.317	83.77	1.43	0.241	17.32	1.62	0.202
	3–4	941 (43.81)	69.53 ± 20.23	—	—	89.37	—	—	25.78	—	—
	≥5	1085 (50.51)	67.34 ± 23.15	—	—	87.34	—	—	27.42	—	—
Household income	<50,000	340 (15.83)	62.27 ± 23.57	5.63	0.018	87.03	1.37	0.253	19.19	7.95	<0.001
	50,000–99,999	948 (44.13)	69.66 ± 21.03	—	—	89.57	—	—	23.77	—	—
	≥100,000	860 (40.04)	68.45 ± 22.64	—	—	86.70	—	—	31.90	—	—
Injured by animals in the past 2 years	No	1977 (92.04)	67.87 ± 22.55	1.24	0.216	87.92	0.08	0.779	27.71	15.27	<0.001
	Yes	171 (7.96)	70.01 ± 18.52	—	—	88.66	—	—	13.50	—	—
Dog ownership	No	1749 (81.42)	67.73 ± 22.56	−1.14	0.257	88.06	0.04	0.838	26.81	0.24	0.627
	Yes	399 (18.58)	69.37 ± 20.78	—	—	87.63	—	—	25.25	—	—
Cat ownership	No	2026 (94.32)	68.06 ± 22.18	0.06	0.951	88.40	4.47	0.035	26.86	1.13	0.287
	Yes	122 (5.68)	67.91 ± 23.19	—	—	81.19	—	—	20.94	—	—
Knowledge score grading	Deficiency	521 (24.26)	39.56 ± 23.02	—	—	71.39	51.19	<0.001	20.18	3.98	0.008
	General	431 (20.07)	66.07 ± 2.27	—	—	88.48	—	—	30.57	—	—
	Good	725 (33.74)	77.32 ± 3.70	—	—	94.62	—	—	29.14	—	—
	Excellent	471 (21.93)	90.24 ± 4.19	—	—	97.56	—	—	26.47	—	—
Attitude	Negative	255 (11.87)	44.49 ± 29.53	—	—	—	—	—	12.23	22.04	<0.001
	Positive	1893 (88.13)	71.27 ± 18.90	—	—	—	—	—	28.46	—	—

### 3.2. Multilevel Model Analysis of KAP Influencing Factors

The three level linear mixed effects model analysis weighted by knowledge scores shows that higher education level, higher household income, animal injury in the past 2 years, and dog ownership are promoting factors for knowledge scores (all *p* < 0.05), while age ≥60 years is an inhibiting factor for knowledge scores (*β* = −7.64, *p* < 0.05). The ICC correlation coefficients within the individual, household, and township levels are 0.371, 0.505, and 0.124, respectively. The analysis of the binomial mixed effects model of attitudes showed that educational level and knowledge score were promoting factors for positive attitudes (all *p* < 0.05; detailed OR and 95% CI are shown in Table 2), with ICC values of 0.255 and 0.173 at the household and township levels, respectively. The binomial mixed effects model analysis of practice showed that dog ownership, high knowledge scores, and a positive attitude were promoting factors for active practice (all *p* < 0.05), while being injured by an animal in the past 2 years was a inhibiting factor for active practice (OR = 0.24, *p* < 0.05). The ICC values at the household and township levels were 0.501 and 0.157, respectively (Table 2).

**Table 2:** Multilevel model analysis of factors influencing rabies KAP of rural residents.

Variables	Classifications	Knowledge score		Attitude		Practice	
		*β* (95% CI)	*p*‐Value	OR (95% CI)	*p*‐Value	OR (95% CI)	*p*‐Value
Fixed effect	Intercept	57.67 (48.78–66.55)	<0.001	8.17 (1.72–38.89)	0.008	0.08 (0.01–0.77)	0.028
County	Changsha	1.00	—	1.00	—	1.00	—
	Liuyang	−0.51 (−7.67 to 6.66)	0.887	0.45 (0.14–1.44)	0.176	1.24 (0.36–4.33)	0.733
	Ningxiang	2.17 (−6.40 to 10.74)	0.609	0.47 (0.13–1.68)	0.243	0.16 (0.03–0.73)	0.018
Gender	Male	1.00	—	1.00	—	1.00	—
	Female	−0.75 (−2.27 to 0.78)	0.338	1.33 (0.93–1.90)	0.119	0.91 (0.63–1.30)	0.592
Age groups	14–17	1.00	—	1.00	—	1.00	—
	18–35	−0.15 (−4.41 to 4.11)	0.946	0.84 (0.36–1.91)	0.670	0.58 (0.19–1.75)	0.330
	36–59	−1.48 (−5.48 to 2.52)	0.468	0.76 (0.36–1.63)	0.482	0.35 (0.12–1.03)	0.057
	≥60	−7.64 (−12.00 to −3.29)	0.001	0.61 (0.27–1.36)	0.227	0.49 (0.16–1.54)	0.222
Education	Primary school and below	1.00	—	1.00	—	1.00	—
	Middle school/high school/vocational school	8.63 (6.06–11.20)	<0.001	1.63 (0.99–2.67)	0.053	0.97 (0.53–1.79)	0.932
	Associate degree	13.98 (10.43–17.52)	<0.001	5.62 (2.19–14.41)	<0.001	1.45 (0.64–3.31)	0.377
	Bachelor’s degree or above	15.02 (11.36–18.69)	<0.001	4.83 (1.83–12.77)	0.001	0.85 (0.36–2.00)	0.709
Household size	1–2	1.00	—	1.00	—	1.00	—
	3–4	−1.56 (−6.34 to 3.21)	0.521	0.76 (0.33–1.75)	0.526	1.87 (0.50–7.01)	0.351
	≥5	−2.71 (−7.43 to 2.01)	0.261	0.63 (0.27–1.43)	0.266	1.52 (0.41–5.61)	0.525
Household income	<50,000	1.00	—	1.00	—	1.00	—
	50,000–99,999	6.65 (3.71–9.59)	<0.001	1.31 (0.72–2.39)	0.376	0.80 (0.41–1.58)	0.526
	≥100,000	6.09 (2.90–9.27)	<0.001	0.93 (0.48–1.81)	0.833	0.63 (0.30–1.30)	0.214
Injured by animals in the past 2 years	No	1.00	—	1.00	—	1.00	—
	Yes	4.73 (1.57–7.89)	0.003	0.85 (0.43–1.69)	0.652	0.24 (0.11–0.53)	<0.001
Dog ownership	No	1.00	—	1.00	—	1.00	—
	Yes	2.61 (−0.01 to 5.23)	0.051	1.13 (0.68–1.88)	0.631	1.90 (1.07–3.36)	0.028
Cat ownership	No	1.00	—	1.00	—	1.00	—
	Yes	−1.13 (−5.31 to 3.05)	0.596	0.55 (0.26–1.19)	0.13	0.67 (0.26–1.74)	0.409
Knowledge score grading	Deficiency	—	—	1.00	—	1.00	—
	General	—	—	3.17 (1.95–5.16)	<0.001	1.98 (1.10–3.56)	0.022
	Good	—	—	7.54 (4.35–13.08)	<0.001	1.85 (1.06–3.26)	0.032
	Excellent	—	—	20.37 (8.66–47.88)	<0.001	1.82 (0.95–3.49)	0.071
Attitude	Negative	—	—	—	—	1.00	—
	Positive	—	—	—	—	3.33 (1.65–6.73)	0.001
Random effect							
Level 1 (Individual)	Variance	166.67	—	3.29^a^	—	3.29^a^	—
	Standard deviation	12.91	—	1.81	—	1.81	—
Level 2 (Household)	Variance	227.28	—	1.47	—	4.81	—
	Standard deviation	15.08	—	1.21	—	2.19	—
Level 3 (Township)	Variance	55.98	—	0.99	—	1.51	—
	Standard deviation	7.48	—	0.99	—	1.23	—

^a^In the multilevel logistic regression model, the individual level residual variance is fixed at *π* ^2^/3 = 3.29 and standardized to 1 at the latent scale. Reference categories for each categorical variable in the multilevel models are those with *β*/OR = 1.00 (e.g., County Changsha, male, etc.).

### 3.3. Analysis of the Mediating Effect of KAP

To examine the mediating role of attitude between knowledge and practice, we established a structural equation model. A significant overall effect linking knowledge to practice was detected (*β* = 0.13, *p* < 0.001). Further decomposition revealed that knowledge significantly positively predicted positive attitudes (*β* = 0.47, *p* < 0.001), and attitudes were in turn positively associated with active practice (*β* = 0.26, *p* < 0.001). Notably, after controlling for attitude, the direct path from knowledge to practice diminished to a nonsignificant level (*β* = 0.01, *p* = 0.778). Attitude emerged as a significant mediator, through which knowledge positively influenced practice (*β* = 0.12, *p* < 0.001). The indirect effect accounts for 91.04% of the overall effect (Figure 1).

**Figure 1:**
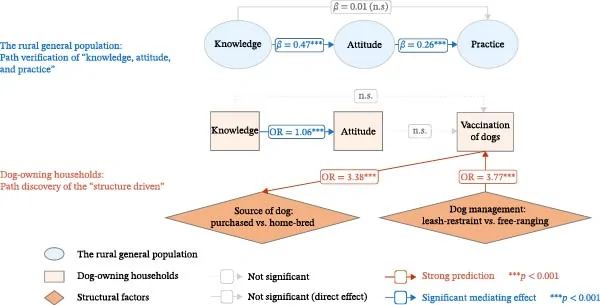
Dual path concept diagram.

### 3.4. Analysis of Vaccination for Domestic Dogs

An analysis of 282 dog‐owning households found that the vaccination rate for domestic dogs was 36.17%. Univariate analysis showed significant differences in the distribution of dog‐vaccination among dog‐owning households in different counties, age groups, educational levels, knowledge score grading, source of dog, and dog management (*χ* ^2^ = 16.52, 10.65, 10.26, 8.70, 18.30, 12.92, *p* < 0.05). The binomial mixed effects model analysis of dog‐vaccination showed that the vaccination rate of purchased dogs was 3.38 times higher than that of home‐bred dogs (OR = 3.38, 95% CI: 1.53–7.45), and the vaccination rate of leash‐restrained dogs was 3.77 times higher than that of free‐ranging dogs (OR = 3.77, 95% CI: 1.84–7.72). The knowledge, attitude, and demographic characteristics of dog‐owning households have no significant predictive effect (Table 3 and Figure 1). The predicted vaccination rate of dogs under the interaction of the source of dog and dog management is shown in Figure 2.

**Figure 2:**
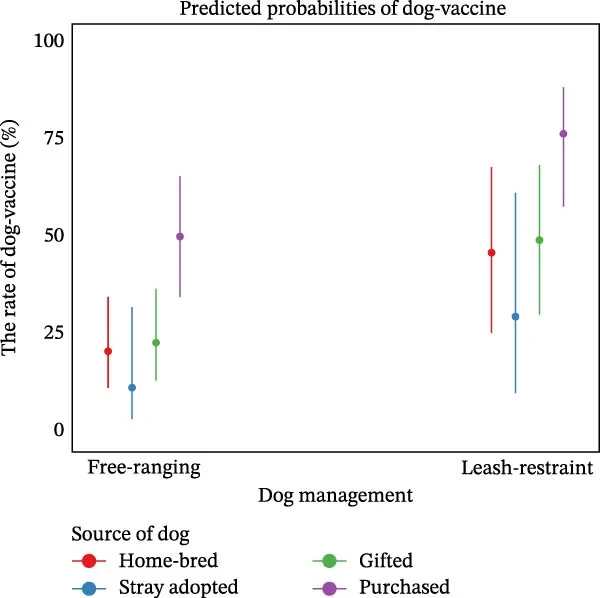
Prediction of dog vaccination rate under the interaction of source of dog and dog management.

**Table 3:** Assessing canine vaccination uptake in dog‐owning households: univariate comparisons and multilevel logistic regression findings.

Variables	Classifications	Univariate analysis				Binomial mixed effects model		
		Number of dog owning households	Vaccination rate of dogs	*χ* ^2^	*p*‐Value	*β*	OR (95% CI)	*p*‐Value
Fixed effect	Intercept	—	—	—	—	−2.90	0.06 (0.01–0.40)	0.004
County	Changsha	111	49.55	16.52	<0.001	—	1.00	—
	Liuyang	53	35.85	—	—	−0.71	0.49 (0.22–1.12)	0.090
	Ningxiang	118	23.73	—	—	−1.13	0.32 (0.15–0.67)	0.002
Gender	Male	144	31.25	2.67	0.103	—	1.00	—
	Female	138	41.30	—	—	0.33	1.39 (0.78–2.46)	0.261
Age groups	14–17	38	26.32	10.65	0.014	—	1.00	—
	18–35	52	36.54	—	—	−0.22	0.80 (0.24–2.70)	0.719
	36–59	134	44.78	—	—	0.47	1.61 (0.60–4.31)	0.347
	≥60	58	22.41	—	—	−0.12	0.88 (0.26–3.00)	0.843
Education	Primary school and below	54	22.22	10.26	0.016	—	1.00	—
	Middle school/high school/vocational school	169	35.50	—	—	0.40	1.50 (0.58–3.89)	0.408
	Associate degree	31	48.39	—	—	0.65	1.92 (0.54–6.87)	0.315
	Bachelor’s degree or above	28	53.57	—	—	1.04	2.83 (0.74–10.86)	0.130
Household size	1–2	19	26.32	1.47	0.479	—	1.00	—
	3–4	130	39.23	—	—	0.86	2.36 (0.66–8.50)	0.188
	≥5	133	34.59	—	—	0.90	2.45 (0.68–8.81)	0.170
Household income	<50,000	62	27.42	2.86	0.239	—	1.00	—
	50,000–99,999	126	37.30	—	—	0.05	1.05 (0.48–2.32)	0.903
	≥100,000	94	40.43	—	—	0.02	1.02 (0.43–2.44)	0.958
Knowledge score grading	Deficiency	73	24.66	8.70	0.033	—	1.00	—
	General	52	30.77	—	—	−0.08	0.92 (0.37–2.32)	0.863
	Good	92	41.30	—	—	0.35	1.41 (0.61–3.27)	0.416
	Excellent	65	46.15	—	—	0.54	1.72 (0.69–4.30)	0.244
Attitude	Negative	34	20.59	3.33	0.068	—	1.00	—
	Positive	248	38.31	—	—	0.42	1.53 (0.52–4.49)	0.440
Injured by animals in the past 2 years	No	216	33.80	1.83	0.176	—	1.00	—
	Yes	66	43.94	—	—	0.22	1.25 (0.64–2.44)	0.522
Source of Dog	Home‐bred	81	29.63	18.30	<0.001	—	1.00	—
	Stray adopted	19	21.05	—	—	−0.89	0.41 (0.11–1.57)	0.193
	Gifted	105	29.52	—	—	0.06	1.06 (0.47–2.38)	0.894
	Purchased	77	55.84	—	—	1.22	3.38 (1.53–7.45)	0.003
Dog management	Free‐ranging	229	31.00	12.92	<0.001	—	1.00	—
	Leash‐restraint	53	58.49	—	—	1.33	3.77 (1.84–7.72)	<0.001
Random effect								
Level 1 (Individual)	Variance	—	—	—	—	3.29^a^	—	—
	Standard deviation	—	—	—	—	1.81	—	—
Level 2 (Township)	Variance	—	—	—	—	0.00	—	—
	Standard deviation	—	—	—	—	0.00	—	—

^a^In the multilevel logistic regression model, the individual level residual variance is fixed at *π* ^2^/3 = 3.29 and standardized to 1 at the latent scale. Reference categories for each categorical variable in the multilevel models are those with OR = 1.00 (e.g., County Changsha, male, etc.).

## 4. Discussion

We assessed the KAP pathway linked to rabies among the general rural population and also explored the key household‐level drivers influencing whether dog owners actually vaccinate their dogs. The results support the KAP framework’s utility in rabies prevention: knowledge provides the initial foundation, but it only translates into appropriate protective actions once a favorable internal attitude has been established. Within the rural population, the indirect effect of knowledge on practice through attitude accounts for over 90% of the overall effect. Nevertheless, the standardized coefficients we obtained fell within the small‐to‐moderate range. This implies that although the hypothesized chain in which knowledge strengthens attitudes and attitudes in turn promote behavioral changes hold true, it likely does not act as the single overwhelmingly dominant mechanism shaping vaccination decisions.

Beyond the individual cognitive framework, various structural obstacles can prevent rural residents from converting favorable attitudes into rabies prevention behaviors. Limited access to both PEP and veterinary services remains a major constraint. Rural areas frequently experience shortages in rabies immunoglobulin and vaccine supply, while veterinary service sites remain widely scattered, so that arranging routine dog vaccination becomes logistically difficult for many households [19, 20]. Financial costs also play a role, including not only the direct expense of vaccination but also indirect items like transport and time lost from work, and these may discourage even risk‐aware dog owners from taking action [21]. In addition, sociocultural influences, such as low perceived threat in communities without recent human cases and persistent misconceptions about domestic dogs safety, can further weaken the attitude‐practice link. These structural factors often neglected in KAP studies may hold the key to closing the gap between what people think and what they actually do.

From a multilevel analysis among dog‐owning households, we found that vaccination status was more closely tied to the dog’s source and management than to the owner’s personal knowledge and attitude. Purchased dogs and leash‐restrained dogs were more likely to be vaccinated. This aligns with earlier reports [22, 23], which noted that bought dogs had higher vaccination odds, whereas dogs obtained as gifts or born at home showed greater nonvaccination risks. The source of a dog may reflect owner investment: purchased dogs tend to receive more deliberate care; home‐born or stray‐adopted dogs often get less attention and therefore require focused public health efforts.

Our findings extend beyond the conventional KAP‐based framework, lending support to a “behavioral opportunities and conditions” perspective. Leash‐restraint is not merely a dog management method but a key condition that creates physical feasibility for vaccine inoculation [24]. Nevertheless, the association between leash‐restraint and vaccination is not uniformly positive. Some work have reported that leash‐restrained dogs may actually receive less routine veterinary care, partly because the owner‐pet attachment tends to be weaker and financial hurdles often limit service use [25, 26]. Such inconsistencies imply that the role of leash‐restraint depends heavily on local contexts. In our study area, where free‐roaming predominates and veterinary services are centralized, leash‐restraint primarily boosts physical accessibility, thereby promoting vaccination.

From a public health standpoint, free‐ranging dogs pose a heightened public health risk. Compared to confined dogs, it is more difficult and costly to vaccinate free‐roaming dogs [13]. Moreover, their unrestricted movement heightens their exposure to rabid wildlife or other infected animals. When these dogs return home, they may carry the rabies virus into the living spaces, thereby raising infection risks for household members, especially young children and older adults. This combination of low vaccination coverage and frequent disease exposure makes free‐ranging dogs a key target for rabies intervention. Given rising global attention to animal welfare, we emphasize that leash‐restraint in this study refers to responsible and humane handling practices. It allows dogs adequate freedom of movement within a defined area, provides sufficient food, water, and shelter, and ensures daily exercise and social interaction. Importantly, it does not mean continuous chaining or confinement. We draw this distinction deliberately, so our proposal is not misread as endorsing inhumane tethering that harms canine welfare.

Our verification of the KAP pathway in the general population and identification of structural drivers among dog‐owning households lead us to propose that rabies prevention should evolve from education‐only approaches toward comprehensive interventions that include dog management reforms, regulation of immunization access in dog trading markets, provision of convenient and affordable vaccination services in communities, and the creation of supportive policy and institutional environments that reduce structural barriers to action. In addition, effective rabies control hinges on an One Health perspective that integrates community education, mass dog vaccination, disease surveillance, healthcare accessibility, and collaborative efforts across veterinary and public health sectors [27].

With respect to study strengths and limitations: our work benefits from a rigorous multilevel modeling approach and a novel analytical perspective that bridges the general population and dog‐owning households. On the other hand, cross‐sectional surveys based on residents’ self‐reports may introduce recall and social desirability biases. Moreover, the generalizability of our findings to other cultural and geographical contexts requires further validation.

## 5. Conclusion

In addition to knowledge and attitude, structural factors—such as dog sources and dog management methods—significantly affect dog vaccination. Rabies prevention should evolve from education toward comprehensive interventions that include dog management reforms and the creation of supportive policy and environments that reduce structural barriers.

## Author Contributions

**Kun Sun:** conceptualization, data curation, formal analysis, investigation, methodology, writing – original draft, writing – review and editing. **Shuilian Chen:** data curation, formal analysis, investigation, methodology. **Chenlin Duan, Jinsong Qiu, Hong Feng, Peng Zhang, and Gangdeng Zheng:** investigation, supervision. **Yinzhu Zhou and Qiangming Xie**: conceptualization, methodology, supervision, writing – review and editing.

## Funding

This research was supported by Hunan Provincial Natural Science Foundation of China (Grant 2026JJ82002).

## Disclosure

All the authors have read and approved the final manuscript.

## Ethics Statement

The questionnaire survey project has obtained ethical approval from the Medical Ethics Committee of Changsha Center for Disease Control and Prevention (CSCDCKYLL2024‐015].

## Consent

Informed verbal consent was obtained prior to participation in the program.

## Conflicts of Interest

The authors declare no conflicts of interest.

## Supporting Information

Additional supporting information can be found online in the Supporting Information section.

## Supporting information


**Supporting Information** Table S1: Questionnaire items used to assess knowledge, attitudes, and practices regarding rabies prevention and control.

## Data Availability

The data supporting the conclusions of this article are included in the article.
