# Supplementary material for: Beyond Knowledge and Attitude: Structural Drivers Dominate Canine Rabies Vaccination Decisions in Rural China: A Multilevel Modeling Study
Source: Transbound Emerg Dis. 2026 Oct 21;2026:4901283. doi: 10.1155/tbed/4901283 (PMC13686368; doi:10.1155/tbed/4901283)
Supplement: Supplementary file 1 [file TBED-2026-4901283-s001.docx]

**Supplementary Material**

**Table S1. Questionnaire items used to assess knowledge, attitudes, and practices regarding rabies prevention and control**

| Dimension | Item No. | Item Content | Response Options |
| --- | --- | --- | --- |
| Knowledge (K) | K1 | Is rabies an infectious disease? | Yes/No/Don‘t know |
|  | K2 | What is the risk of death after contracting rabies in humans? | None/Low/Middle/High/Definitely die/Don‘t know |
|  | K3 | Which animals do you think may carry rabies virus? (Multiple Choice) | Dogs/Cats /Poultry such as chickens and ducks/ Wild animals/Bats/Don‘t know |
|  | K4 | Which of the following ways may lead to human infection with rabies? (Multiple Choice) | Bare skin lightly bitten by animals, or minor scratches or abrasions without obvious bleeding;  Skin bitten or scratched (with bleeding);  Damaged skin being licked;  Open wounds, mucous membranes contaminated with saliva or tissue;  Direct contact with bats;  None of the above will be infected;  Don‘t know; |
|  | K5 | Do you think rabies can be prevented? | Sure/Can not/Don‘t know |
|  | K6 | Is it necessary to treat wounds after being injured by animals? | Necessary/Not Necessary/Don‘t know |
|  | K7 | How to deal with wounds caused by animals?(Multiple Choice) | Squeeze the wound;  Wash the wound alternately with soapy water and water of a certain pressure for at least 15 minutes;  Disinfect the wound with disinfectant;  Bandaging to stop bleeding;  Don‘t know |
|  | K8 | Is it necessary to get vaccinated after being injured by animals? | Necessary/Not Necessary/Don‘t know |
|  | K9 | What is the best time to get vaccinated after being injured by an animal? | Immediately, the earlier the better/Within 24 hours /Within 3 days/Don‘t know |
|  | K10 | Is it necessary to inject immunoglobulin if there is bleeding from the wound? | Necessary/Not Necessary/Don‘t know |
|  | K11 | If injected with rabies virus vaccine, can immunoglobulin be dispensed with? | Yes/No/Don‘t know |
|  | K12 | Can the meat of a dog that has injured a person be eaten after being hunted and killed? | Yes/No/Don‘t know |
| Attitude (A) | A1 | Rabies poses a significant threat to human health | Agree/uncertain/disagree |
|  | A2 | When encountering stray dogs, you should report to the police station for handling | Agree/uncertain/disagree |
|  | A3 | Dog owners should register their dogs for standardized management | Agree/uncertain/disagree |
|  | A4 | Dog owners should walk their dogs with ropes to restrict their movement and avoid harming others | Agree/uncertain/disagree |
|  | A5 | People who own dogs/cats should regularly vaccinate them against rabies to prevent the spread of rabies | Agree/uncertain/disagree |
|  | A6 | Even seemingly healthy animals need to be vaccinated against rabies | Agree/uncertain/disagree |
|  | A7 | People who own dogs/cats and have not been bitten or scratched can also receive rabies vaccine in advance to prevent rabies | Agree/uncertain/disagree |
|  | A8 | After being injured by animals, wound treatment and vaccination should be carried out as soon as possible | Agree/uncertain/disagree |
|  | A9 | After being injured by vaccinated animals, the injured still need to be vaccinated | Agree/uncertain/disagree |
|  | A10 | After receiving the rabies vaccine, if a person is injured again by an animal, they also need to go to the clinic to treat the wound and consult whether they need to receive a booster shot. | Agree/uncertain/disagree |
| Practice (P) | P1 | Teasing one's own or someone else's pet | Often /occasionally /never |
|  | P2 | Feeding or teasing stray animals | Often /occasionally /never |
|  | P3 | Drive away and capture stray animals | Often /occasionally /never |
|  | P4 | Provoking animals that are eating, sleeping, or protecting their cubs | Often /occasionally /never |
|  | P5 | Do not allow animals to lick wounds on the hands | Often /occasionally /never |
|  | P6 | After learning that people around them have been injured by animals, they will be advised to get vaccinated | Often /occasionally /never |
| Covariates | C1 | Gender | Male/ Female |
|  | C2 | Age | _____ years old |
|  | C3 | Educational level | Primary school or below/ Junior high school/ High school or vocational school/ Junior college/ Undergraduate/ Master's degree or above |
|  | C4 | Monthly average income level | ≤ 1000 yuan/ ≤ 3000 yuan/ ≤ 5000 yuan/≤ 10000 yuan/ Above 10000 yuan |
|  | C5 | Residential districts and counties: | Changsha County /Liuyang City /Ningxiang City |
|  | C6 | The street or township where it is located | fill in the blank |
| Dog related variables | D1 | Source of dogs: | Purchase /Gift from others /Adoption of stray dogs  / Children born to their own dog/Other |
|  | D2 | Dog management | Free-ranging/ leash-restrained |
|  | D3 | Has the dog been vaccinated against rabies? | Yes/No |

(1) Knowledge：Calculate the total number of correctly answered questions and linearly convert them into standardized continuous variables ranging from 0 to 100 points, with higher scores indicating richer knowledge.

(2) Attitude: Using a 3-point Likert scale rating (agree=3, unsure=2, disagree=1). All items are positive statements, and the total score is converted to 0-100 points after standardization. For binary classification analysis, based on the sample distribution of this study, a standardized score of ≥ 85 is defined as "positive attitude", while the rest are defined as "negative attitude".

(3) Practice: With 4 items assessing risky behavior (reverse scoring: never=3, occasionally=2, frequently=1) and 2 items assessing protective behavior (positive scoring: frequently=3, occasionally=2, never=1). The total score has been standardized to 0-100 points. According to the method of dividing attitude variables, a standardized score of ≥ 85 is defined as "active practice", while the rest are defined as "inactive practice".

(4) Covariance: The collected social demographic characteristics (such as age, gender, education level, household income) and the classification of dog related variables, such as source of dog, dog management, are detailed in the subsequent analysis results table.
